# Genistein promotes cell death of ethanol-stressed HeLa cells through the continuation of apoptosis or secondary necrosis

**DOI:** 10.1186/1475-2867-13-63

**Published:** 2013-06-26

**Authors:** Xin Xie, Shan Shan Wang, Timothy Chung Sing Wong, Ming Chiu Fung

**Affiliations:** 1 School of Life Sciences, the Chinese University of Hong Kong, Room EG07, Shatin, New Territory, Hong Kong, SAR, China

**Keywords:** Genistein, HeLa Cell, Stress Recovery, Apoptosis, Necrosis

## Abstract

**Background:**

Apoptosis is a major target and treatment effect of multiple chemotherapeutical agents in cancer. A soybean isoflavone, genistein, is a well-studied chemopreventive agent and has been reported to potentiate the anticancer effect of some chemotherapeutics. However, its mechanistic basis of chemo-enhancement effect remains to be fully elucidated.

**Methods:**

Apoptotic features of low concentration stressed cancer cells were studied by microscopic method, western blot, immunostaining and annexin V/PI assay. Genistein’s effects on unstressed cells and recovering cells were investigated using MTT cell viability assay and LDH cytotoxicity assay. Quantitative real-time PCR was employed to analyze the possible gene targets involved in the recovery and genistein’s effect.

**Results:**

Low-concentration ethanol stressed cancer cells showed apoptotic features and could recover after stress removal. In stressed cells, genistein at sub-toxic dosage promoted the cell death. Quantitative real-time PCR revealed the up-regulation of anti-apoptotic genes *MDM2* and *XIAP* during the recovery process in HeLa cells, and genistein treatment suppressed their expression. The application of genistein, MDM2 inhibitor and XIAP inhibitor to the recovering HeLa cells caused persistent caspase activity and enhanced cell death. Flow cytometry study indicated that genistein treatment could lead to persistent phosphatidylserine (PS) externalization and necrotic events in the recovering HeLa cells. Caspase activity inhibition shifted the major effect of genistein to necrosis.

**Conclusions:**

These results suggested two possible mechanisms through which genistein promoted cell death in stressed cancer cells. Genistein could maintain the existing apoptotic signal to enhance apoptotic cell death. It could also disrupt the recovering process in caspase-independent manner, which lead to necrotic events. These effects may be related to the enhanced antitumor effect of chemotherapeutic drugs when they were combined with genistein.

## Background

Conventional chemotherapeutic agents were screened or developed based on the feature that tumor cells usually proliferate faster than normal cells [1,2]. By interacting with different intracellular targets such as DNA molecules or microtubules, chemotherapeutics introduce various degrees of damage or stress in rapidly dividing cells, leading to toxic effects [3,4]. Although the molecular details responsible for their anti-cancer effects were not fully unveiled, most anti-cancer drugs exert their toxicity through apoptotic cell death [3-5]. The significance of apoptosis in tumorigenesis and chemotherapy is further underpinned by frequent mutations of genes involved in apoptotic initiation and execution, and defects in apoptosis may impair treatment effect [6,7]. The findings that chemo-resistance of cancer cells is often associated with defective apoptosis makes the restoration of effective apoptosis a potential therapeutic improvement, either through suppressing anti-apoptotic functions or restoring the pro-apoptotic activities [8,9].

Genistein is a predominant soybean isoflavone whose anti-cancer effects have been intensively studied. Epidemiological studies suggested that the lower incidence of prostate and breast cancer in Asian, when compared to western countries, could be attributed to the rich isoflavonoid content found in Asian diets-including genistein [10-12]. Both *in vitro* and *in vivo* analyses showed that genistein inhibits the growth of various cancer cells at doses non-toxic to normal cells [13-15]. In terms of its known molecular targets in cancer cells, genistein was proved to be a competitive inhibitor for estrogen receptor, interfere signal transduction by inhibiting the activity of protein tyrosine kinase, suppress angiogenesis, and arrest cell cycle at G_2_-M transition [16-18]. The investigation of gene expression profile of genistein-treated prostate cancer cells also revealed that genistein could regulate the expression of genes involved in various cell processes such as proliferation, cell cycle progression, transcription, apoptosis, oncogenesis, angiogenesis, and cancer cell invasion and metastasis [19,20]. Besides, increasing number of research indicated that genistein promoted the anti-tumor effect of chemotherapeutics towards multiple types of tumors, implying that genistein could be a useful chemopreventive agent [21-23]. However, the mechanistic basis for its chemo-enhancement effect remains to be fully characterized.

In this study, we investigated the effect of genistein on low concentration ethanol- stressed HeLa cells with apoptotic features. Stressed HeLa cells could recover after replacing the ethanol-containing medium with fresh medium. We found that genistein promoted the cell death of stressed HeLa cells at the concentration without detectable toxicity to untreated cells. The death-promoting effect might result from the suppression of anti-apoptotic genes including *MDM2* and *XIAP*, which were up-regulated during recovery process after stress removal. Similar to genistein’s effect, the inhibition of XIAP and MDM2 protein activity resulted in persistent caspase-3 activity and enhanced cell death in the recovering HeLa cells. Nevertheless, the application of caspases inhibitor did not rescue the recovering cells from the death-promoting effect of genistein. Collectively, our data provided evidence on the death-potentiating effect of genistein on stressed HeLa cells through caspase- dependent or independent way.

## Results

### Low concentration ethanol stressed HeLa cells displayed apoptotic features and the stressed cell could recover after stress removal

We initially aimed to set up stressed condition that could initiate apoptotic features in HeLa cells. Ethanol-induced hepatic apoptosis has been widely reported in experimental trials with different animals and clinical alcoholic diseases [24]. However, the toxic effects of ethanol correlate with the applied concentrations and exposure time. High concentrations of ethanol could cause necrotic cell death [25]. Low concentration ethanol treatment was reported to induce apoptosis in HepG2 cells, and both fas- and cytochrome C-mediated pathway may be involved [24,25]. In human intestinal Caco-2 cell line, treatment with 10% ethanol up to 3 hours could lead to considerable PS externalization. Caspase-mediated CK18 cleavage and DNA fragmentation was detected after 2 hours’ treatment [26].

**Figure 1:**
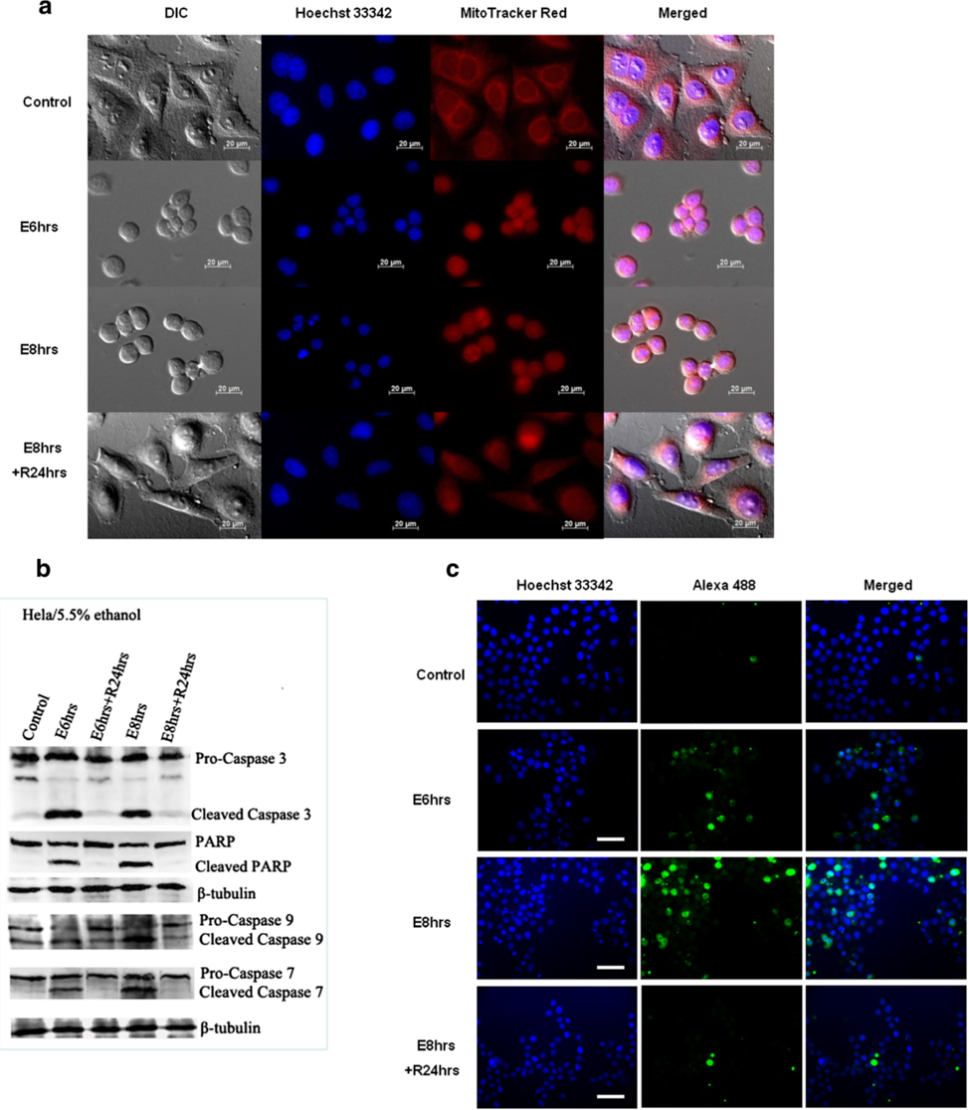
**Low concentration ethanol stressed HeLa cells displayed apoptotic features but could recover after stress removal.** (**a**). Live cell imaging of HeLa cells before stress treatment (control), 5.5% ethanol stressed for 6 (E6 hrs) and 8 h (E8 hrs), and after stress removal for 24 h (R24 hrs). Mitochondria (red, MitoTracker Red Staining) and Nuclei (blue, Hoechst 33342 staining) were visualized by fluorescence (Scale bar: 20 μm). (**b**). Western blot was used to detect the cleavage of PARP and caspase enzymes after stress treatment (E6 hrs & E8 hrs) and 24 h recovery (E6 hrs + R24 hrs & E8 hrs + R24 hrs). (**c**). Immunostaining of cleaved caspase-3 of stressed (E) and recovered (E + R) HeLa cells. After incubated with anti-cleaved caspase 3 antibody, the cells were further stained with Alexa Fluor® 488-conjugated secondary antibody. Nuclei were stained with Hoechst 33342 and the cells were visualized with fluorescence microscopy (Scale bar: 100 μm).

We optimized the ethanol (stress) concentration and exposure time used to stress HeLa cells. Incubation in ethanol-containing medium (5.5% , V:V) up to 8 hours (E8hrs) caused significant cellular shrinkage while the shrunken cells regained normal morphology after replacing stressed medium with fresh culture medium (E8 hrs + R24 hrs) (Figure 1a). Nuclear condensation and the disruption of mitochondrial network were observed, which were reversible after the removal of stress (Figure 1a).

**Figure 2:**
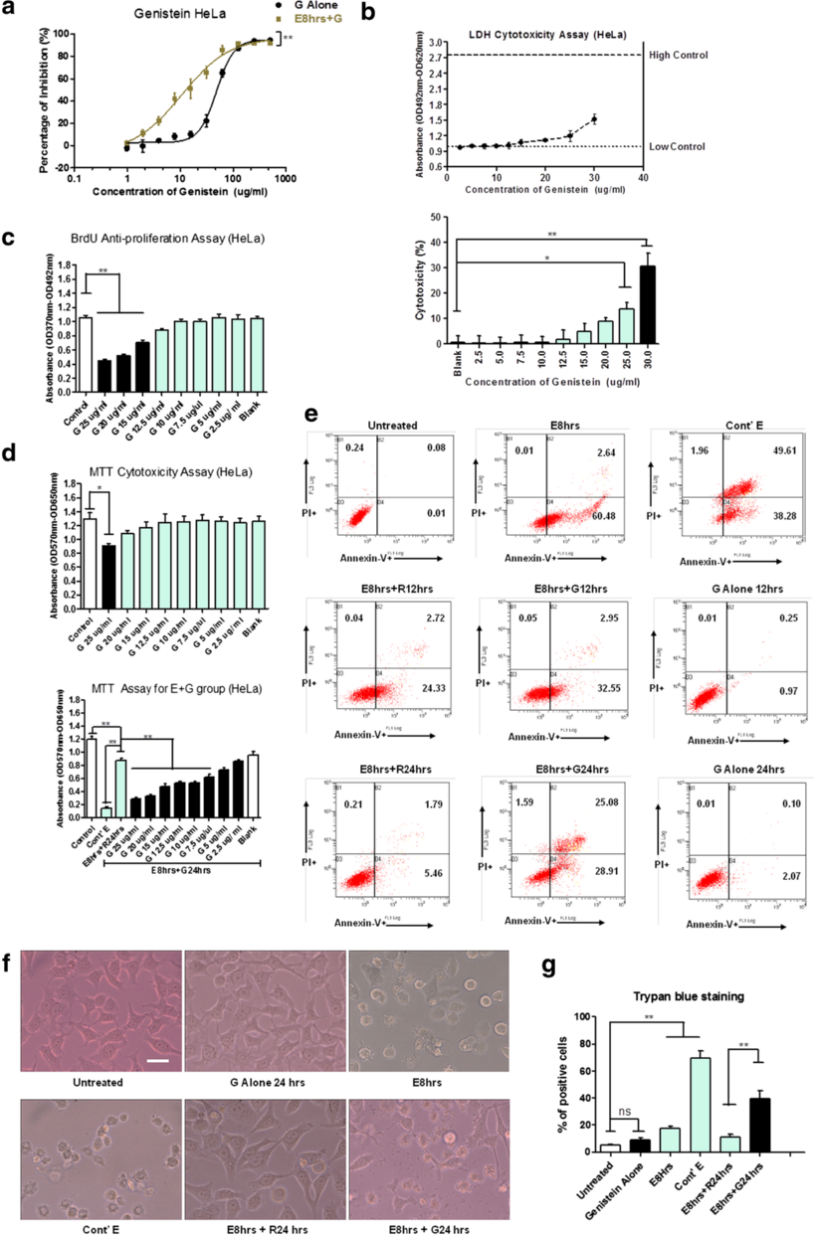
**Genistein promoted cell death of stressed cancer cells at sub-toxic concentration to unstressed cells.** (**a**). Dose-dependent responsive curves of genistein on unstressed cells (G Alone) and stressed cells (E + G) of HeLa for 48 h treatment by MTT assay. Percentage of inhibition was calculated as: [(mean value of untreated control – each value of G alone group)/ mean value of untreated control] x 100% for Genistein Alone (G Alone) group; [(mean value of stressed + fresh medium (E + R) readings – each value of stressed + genistein (E + G) group)/ mean value of stressed + fresh medium (E + R) readings] x 100% for stressed + genistein (E + G) group. Two-way ANOVA was used to test the significance of ethanol stress as the source of variance. (**b**). LDH cytotoxicity assay of genistein treatment on HeLa cell for 18 h. Upper figure: LDH assay reading of dose-dependent genistein treatment: low control: untreated sample; High control: Triton X-100 treated. Bottom figure: percentage of cytotoxicity of dose-dependent genistein treatment. (**c**). BrdU cellular proliferation assay of dose-dependent genistein treatment of HeLa cells for 48 h. G stands for genistein. (**d**). Dose-dependent effects of genistein on untreated HeLa cell (upper figure) and ethanol-stressed HeLa cells (E8 hrs + G24 hrs, lower figure) for 24 h. Cont’ E: continuous ethanol stress treatment; E8 hrs + R24 hrs: ethanol stressed for 8 h and ethanol was replaced by fresh medium for 24 h recovery; Blank: DMSO solvent without genistein. (**e**). Annexin V and propidium iodide staining of HeLa cells before (untreated) and after stress treatment (E8 hrs), and after the removal of stress (E8 hrs + RXhrs). Groups treated with genistein (15 μg/ml) alone (G Alone) and the stressed groups treated with genistein (15 μg/ml) after stress removal (E8 hr + GXhrs) were included. Cont’ E for continuous ethanol stress treatment group. The percentage of events of early apoptotic (lower right), and late apoptotic or necrotic (upper right) is indicated in each diagram. (**f**). Morphological changes of HeLa cells after stress treatment (E8hrs), stress treatment and 24 h recovery (E8 hrs + R24 hrs), and recovery in presence of genistein (15 μg/ml) (E8 hrs + G24 hrs) under inverted phase contrast microscope (Scale bar: 10 μm). (**g**). Trypan blue dye exclusion assay of HeLa cells after stress treatment, stress treatment and 24 h recovery, and recovery in presence of genistein (15 μg/ml). (Data points in above figures represent Mean ± SEM of three independent experiments; SEM = error bar; * indicates: *p* < 0.05; ** indicates: *p* < 0.01).

In addition to morphological changes, biochemical markers of apoptosis were investigated. Western blot and caspase-3 immunostaining results showed the cleavage of biochemical marker caspase-3 and its substrate PARP in the stressed cells (Figure 1b, c: E6 hrs & E8 hrs). The activation of caspase-7 and −9 were also detected (Figure 1b). The disappearance of cleaved caspases and PARP after 24 hours’ recovery (E8 hrs + R24 hrs) indicated that apoptotic signals could be terminated and eliminated in the absence of continuous stress. Furthermore, Annexin V and PI staining assay revealed the loss of membrane asymmetry after stress treatment in the form of phosphatidylserine (PS) externalization (Figure 2e, E8 hrs), and the gradual restoration of membrane asymmetry after stress removal (Figure 2e, E8 hrs + R12 hrs & E8 hrs + R24 hrs). The gradual increase of annexin V signal intensity with the ethanol stress time was consistent with previous study concerning ethanol-induced apoptosis in Caco-2 cells [26], which seems to be a concentration- and time-dependent process. Collectively, our results demonstrated the ethanol-stressed apoptotic features, including morphological and biochemical alterations, were reversible in HeLa cells. The optimized stress conditions (5.5% ethanol 8 hrs for HeLa), on which most cells displayed reversible changes, were used for the following study.

### At sub-toxic concentration, genistein promoted the cell death of stressed HeLa cells after the removal of ethanol

Based on the optimized stress conditions, we compared the dose-responsive curves of genistein between stress and unstressed cells using MTT assay. Stressed HeLa cells were more sensitive to genistein treatment than unstressed ones, manifested by the left shift of dose–response curve of stressed group (Figure 2a, *p* < 0.01 by two-way ANOVA) and its lower IC50 value (concentration of genistein with 50% inhibitory effect: 48.55 μg/ml for unstressed cells; 10.53 μg/ml for stressed cells). To exclude the effect of direct cytotoxicity and anti-proliferation of genistein, we then detected the cytotoxic range of genistein by lactate dehydrogenase release assay and its effective dose of anti-proliferation effect by BrdU incorporation assay. The death-promoting effect of genistein on stressed HeLa cells existed at the concentration (10 μg/ml) where there were no detectable cytotoxicity to unstressed group (Genistein ≥ 25 μg/ml to be cytotoxic in HeLa cells) and anti-proliferative effects (Genistein ≥ 15 μg/ml to show detectable anti-proliferation effect in HeLa cells) (Figure 2b, c, d). Below the threshold of cytotoxicity, 10 μg/ml genistein treatment significantly reduced MTT reading of stressed cells, compared to the MTT reading of stressed cells with fresh medium replacement only (Figure 2d, *p* < 0.01).

We further applied Annexin-V and PI staining assay to investigate genistein’s effect quantitatively. Flow cytometry results demonstrated that genistein treatment (at concentration 15 μg/ml, no cytotoxicity effect) inhibited the membrane asymmetry restoration of stressed cells (E8 hrs + G12 hrs & E8 hrs + G24 hrs), while genistein treatment on unstressed cells showed no significant effect (G Alone 24 hrs) (Figure 2e). In the presence of genistein (E8 hrs + G24 hrs), Annexin V single-positive cells persisted and the percentage of Annexin V/PI double-positive cells increased in the stressed cell population, indicating pleiotropic effects of genistein on the recovery from stress. The cellular morphologies in different experimental groups were showed in Figure 2f, which was consistent with the flow cytometry data to show the death-promoting effect of genistein on the stressed cells. Trypan blue dye exclusion assay was used to quantify the terminally dead cells which are permeable to the dye. The results further verified that the genistein promoted the death of stressed cells which was undergoing the recovery process (Figure 2g).

### Genes involved in recovery of stress treatment were influenced by genistein

**Figure 3:**
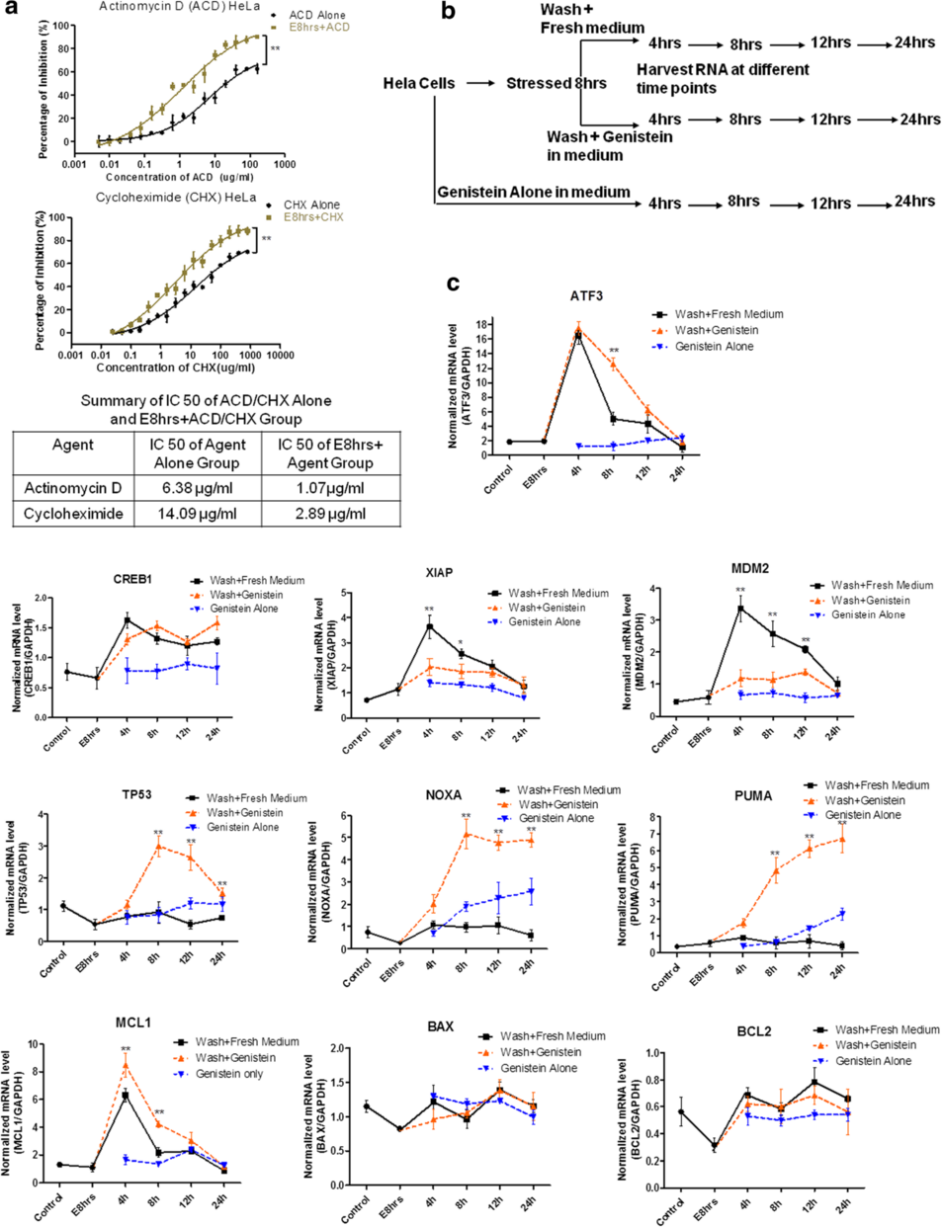
**Genes involved in the recovery from stress treatment were influenced by genistein.** (**a**). Dose-dependent responsive curves of Actinomycin D and Cycloheximide on unstressed cells (ACD Alone and CHX Alone) and stressed cells (E8 hrs + ACD and E8 hrs + CHX) of HeLa for 48 h treatment by MTT assay. Two-way ANOVA was used to test the significance of ethanol stress as the source of variance. IC50 of each group is summarized in table. (**b**). Schematic schedule of RNA sample collection for each treatment group, including genistein treatment alone, stressed and replaced with fresh medium for recovery, and stressed and replaced with genistein-containing medium. (**c**). Time-dependent gene expression profiles of *ATF3*, *CREB1*, *XIA*P, *MDM2*, *TP53*, *NOXA*, *PUMA*, *MCL1*, *BAX*, *BCL2*, in different groups of HeLa cell. Control stands for the untreated cells; E8 hrs represents ethanol-stressed cells for 8 hrs; Black line: ethanol-stressed and stress replaced with fresh medium; Orange line: ethanol-stressed and stress replaced with medium containing 15 μg/ml genistein. Blue line: unstressed cells treated with 15 μg/ml genistein. Each gene expression level is normalized to that of GAPDH. (Data point above is expressed as Mean ± SEM, n = 3; asterisk indicates statistical comparison of each time point between Wash + Fresh Medium and Wash + Genistein group by tow-way ANOVA, * indicates: *p* < 0.05; ** indicates: *p* < 0.01).

To study the mechanism of the recovery from stress and the death-promoting effect of genistein, we first compared the sensitivity difference to transcription and translation inhibitor (Actinomycin D and Cycloheximide) between stress and unstressed HeLa cells. Dose-responsive curve indicated stressed cells undergoing recovery process were more sensitive to transcription and translation inhibition (Figure 3a, *p* < 0.01 by two-way ANOVA). We then collected mRNA samples of HeLa cells at different time points after the removal of stress in the absence or presence of genistein, including the group treated with genistein alone (Figure 3b). Quantitative real-time PCR was applied to determine the mRNA level of genes relevant to apoptosis or survival. Among the genes investigated, *ATF3*, *CREB1*, *XIAP*, *MDM2* and *MCL1* were involved in the recovery period, which were up-regulated after the stress removal and then gradually returned to the level of untreated control (Figure 3c, statistical comparison of each time point between Wash + Fresh Medium and Wash + Genistein group by tow-way ANOVA, * indicates: *p* < 0.05; ** indicates: *p* < 0.01). Genistein treatment interfered with the expression profiles of *ATF3*, *XIAP*, *MDM2* and *MCL1* genes during recovery phase. Genistein delayed the decrease of *ATF3* and *MCL1* expression, but attenuated up-regulation of *XIAP* and *MDM2* in the group undergoing recovery, while genistein treatment alone to unstressed group did not have that effect (Figure 3c). Other genes including *TP53*, *NOXA*, *PUMA* did not seem to be regulated during the recovery period after stress removal. However, genistein treatment dramatically increased their expression levels after the removal of stress. The third group of genes such as *BCL2* and *BAX* did not seem to play significant part in the recovery and in the genistein treatment group. Overall, the gene expression profiles demonstrated differential gene expressions in the recovery from stress treatment and the influence of genistein on their regulations. *XIAP* and *MDM2* genes, whose expressions were up-regulated in the recovery process and attenuated in the presence of genistein, were subject to further investigation.

### Similar to genistein, MDM2 and XIAP inhibitor potentiated the cell death and caused persistent caspase-3 activity in recovering cells

**Figure 4:**
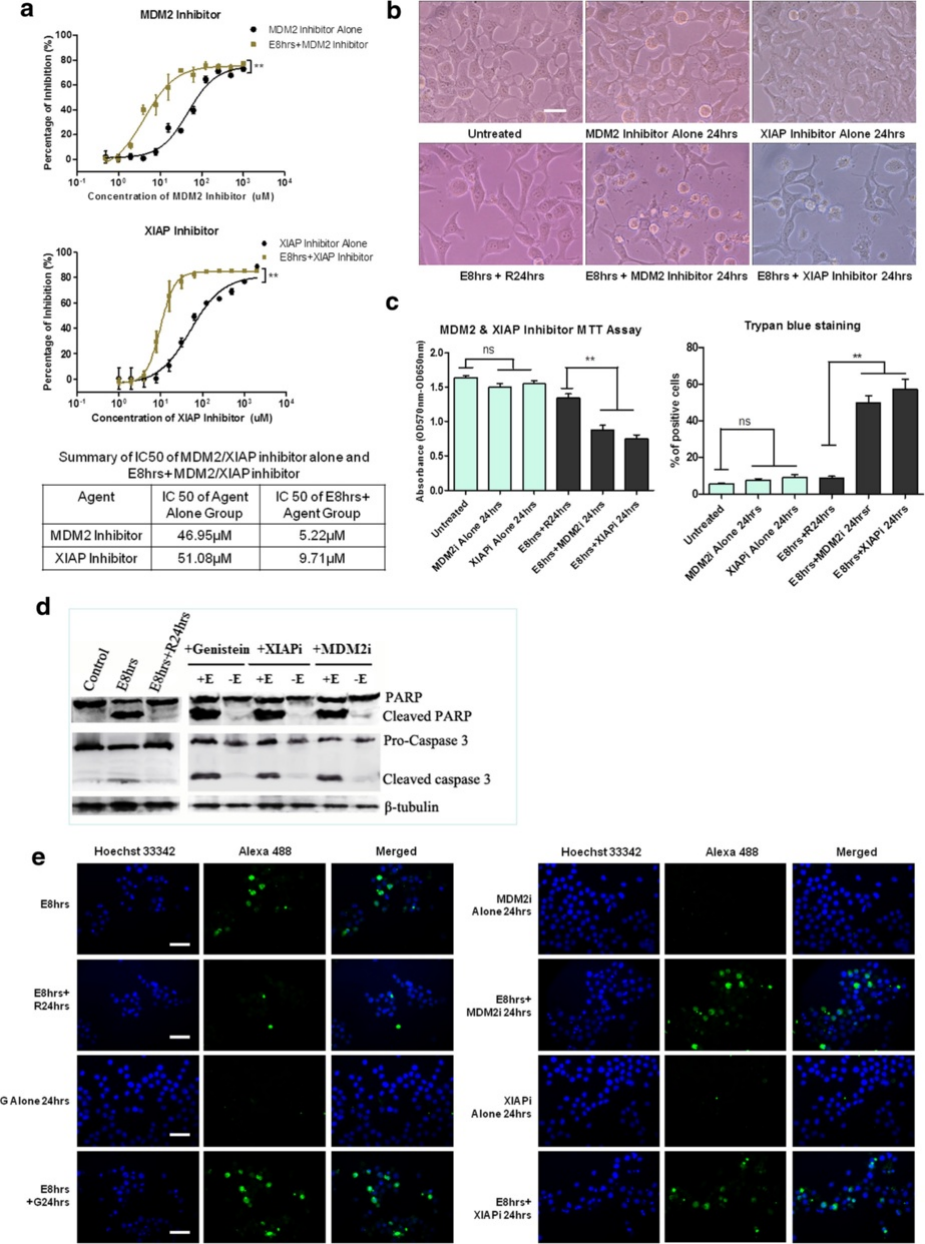
**MDM2 and XIAP inhibitor potentiated the cell death and caused persistent caspase activity in stressed HeLa cell.** (**a**). Dose-dependent responsive curves of MDM2 inhibitor (Boranyl-chalcone) and XIAP inhibitor (Embelin) on unstressed cells (Inhibitor Alone) and stressed cells (E8 hrs + Inhibitor) of HeLa for 48 h treatment by MTT assay. Two-way ANOVA was used to test the significance of ethanol stress as the source of variance. IC50 of each group is summarized in table. (**b**). Morphology of HeLa cells recovered 24 h from stress (E8 hrs + R24 hrs) and cells treated with MDM2 inhibitor (10 μM) or XIAP inhibitor (20 μM) for 24 h during recovery period (E8hrs + inhibitor 24 hrs). Inhibitor treatment alone for 24 h was included (Scale bar: 10 μm). (**c**). Cell viability assay of the effects of MDM2 inhibitor (10 μM) or XIAP inhibitor (20 μM) on HeLa cells undergoing recovery from stress treatment for 24 h treatment. Left: MTT cell viability assay; Right: Trypan blue dye exclusion assay (Mean ± SEM, n = 3; ns indicates: no significance; ** indicates: *p* < 0.01). XIAPi stands for XIAP inhibitor; MDM2i stands for MDM2 inhibitor. (**d**) Western blot analysis of the effects of genistein (15 μg/ml), MDM2 inhibitor (10 μM) and XIAP inhibitor (20 μM) 24 h treatment on HeLa cells undergoing stress recovery. +E: 5.5% ethanol stress for 8 hrs followed by genistein and inhibitor treatment. -E: without ethanol stress treatment. (**e**). Immunostaining of cleaved caspase-3 of stressed and unstressed HeLa cells treated with genistein (15 μg/ml), MDM2 inhibitor (10 μM) and XIAP inhibitor (20 μM). Cleaved caspase-3 (Green) stained with Alexa Fluor® 488-conjugated antibody, Nuclei (Blue) stained with Hoechst 33342 (Scale bar: 100 μm).

To confirm the roles of MDM2 and XIAP in the recovery of stressed cells, we first compared the sensitivity of stressed cells and unstressed cells to MDM2 inhibitor (Boranyl-chalcone) and XIAP inhibitor (Embelin). IC50 difference (Figure 4a, table) indicated that HeLa cells undergoing recovery are highly dependent on MDM2 and XIAP activity. XIAP and MDM2 inhibitor treatment suppressed the regaining of normal morphology of stressed cells after stress removal (Figure 4b). MTT and Trypan blue assay demonstrated that XIAP and MDM2 inhibition caused further loss of cellular viability and enhanced cell death to the recovering cells at the concentration where there were no significant effects to unstressed cells (Figure 4c). More importantly, western blot and immunostaining results revealed that after the removal of stress, apoptotic marker like cleaved caspase 3 and PARP persisted in the recovering HeLa cells in the presence of genistein, XIAP and MDM2 inhibitor (Figure 4d, e). These data suggested that genistein might enhance the cell death of stressed HeLa cells by continuing the existing apoptotic signal or by preventing the clearance of activated caspases, possibly through the interference with MDM2 and XIAP expression.

### The death-promoting effect by genistein could be caspase-independent

**Figure 5:**
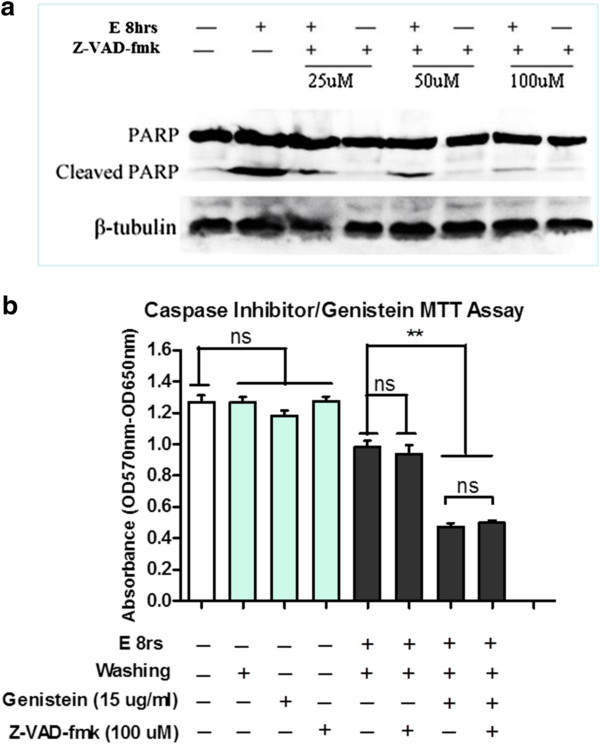
**Caspase activity inhibition did not rescue the effects of genistein’s effect on recovering HeLa cells.** (**a**). HeLa cells were stressed or unstressed with 5.5% ethanol for 8 h (E8 hrs), in the absence (−) or the presence (+) of 25, 50, and 100 μM of general caspase inhibitor Z-VAD-fmk, and the PARP cleavage was examined by Western blot. (**b**). MTT assay about genistein’s effects on stressed HeLa cell, in the absence (−) or the presence (+) of general caspase inhibitor Z-VAD-fmk (100 μM) for 24 h. E 8 hrs stands for 8 h ethanol stress; Washing stands for the removal of ethanol-containing medium (Data expressed as Mean ± SEM, n = 3; ns indicates: no significance; ** indicates: *p* < 0.01).

Now that apoptotic signal like cleaved caspase 3 persisted in the recovering cells when genistein was present, we further applied general caspase inhibitor to investigate whether the caspase activity inhibition could rescue genistein’s death-enhancing effect on the stressed HeLa cells. General caspase inhibitor Z-VAD-fmk effectively blocked caspase activity at the concentration of 100 μM, manifested by the great reduction of cleaved PARP (Figure 5a). Through MTT viability assay, the addition of Z-VAD-fmk did not rescue the death-promoting effects of genistein on the recovering cells, which indicated that genistein’s effect was not restricted to the enduring and potentiation of apoptotic signal (Figure 5b).

### Caspase inhibition shifted genistein’s action profile

**Figure 6:**
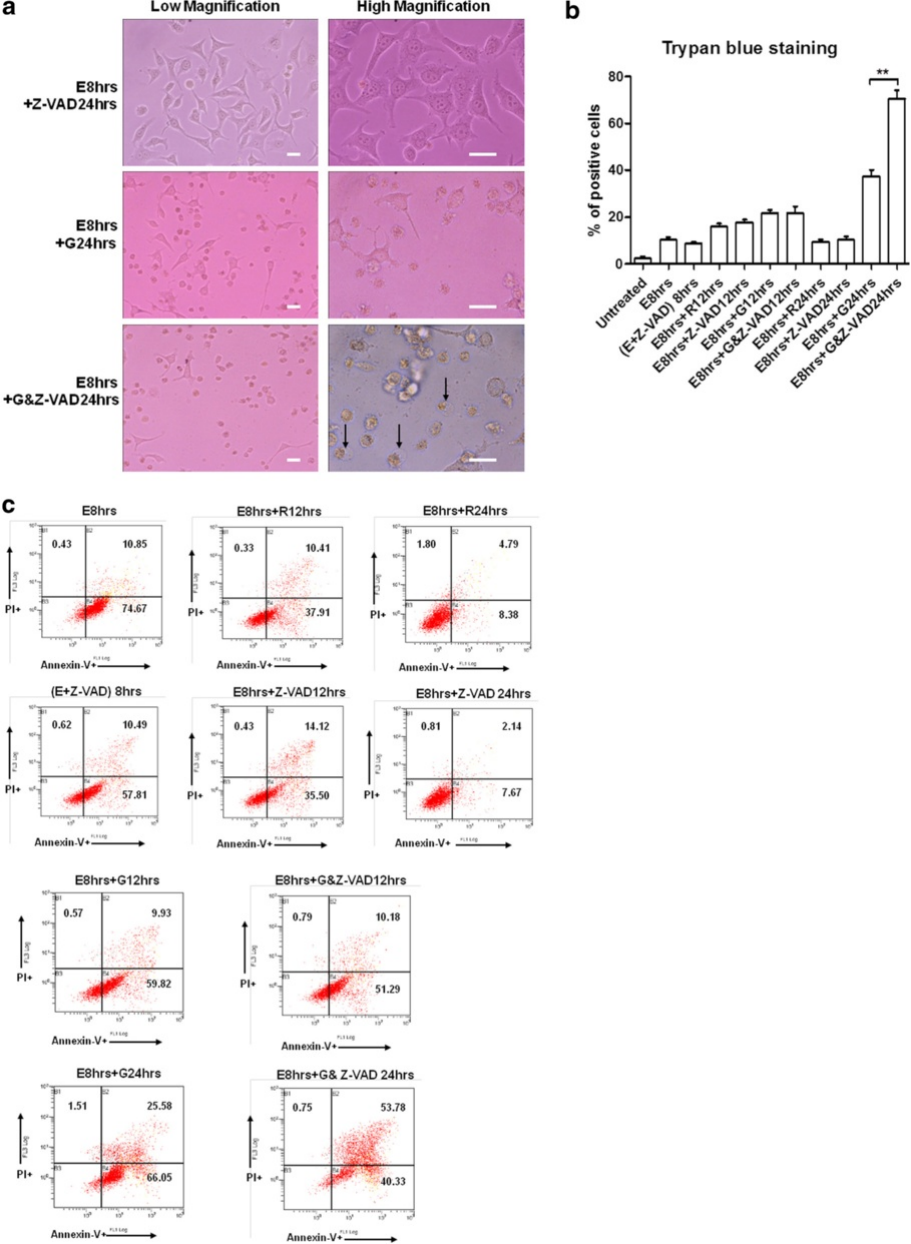
**Caspase activity inhibition shifted the effects of genistein to secondary necrosis.** (**a**). Morphology of HeLa cells undergoing the recovery from stress treatment, in the presence of 100 μM Z-VAD-fmk (upper), or 15 μg/ml genistein (middle), or both (bottom) for 24 h. Arrows indicate the cell swelling and membrane rupture of shrunk cells. (**b**). Trypan blue dye exclusion assay of HeLa cells after the removal of stress, in the presence of 15 μg/ml genistein or 100 μM Z-VAD-fmk or both for 12 h and 24 h respectively (Data expressed as Mean ± SEM, n = 3; ** indicates: *p* < 0.01). (**c**). Annexin V and propidium iodide staining assay of HeLa cells after stress treatment and stress removal in the presence of 15 μg/ml genistein or 100 μM Z-VAD-fmk or both for 12 h and 24 h respectively. (E + Z-VAD) 8 hrs stands for ethanol stress for 8 h in the presence of Z-VAD. The percentage of events early apoptotic (lower right), and late apoptotic or necrotic (upper right) were indicated in each diagram.

Another question to be considered was what are other factors contributing to genistein’s effect in the recovering HeLa cells? Using Annexin V/PI staining assay, we observed that caspase activity inhibition contributed little to the recovery process after the stress removal (Figure 6c). However, the addition of caspase inhibitor significantly altered the profile of genistein’s effect by a great percentage shift from Annexin V single positive cells to Annexin V/PI double-positive cells after 24 hours (Figure 6c: E8 hrs + G24 hrs & E8 hrs + G&Z-VAD24 hrs). These changes were further manifested by the increase of cells displaying necrotic features in the presence of both genistein and Z-VAD-fmk (Figure 6a: E8 hrs + G&Z-VAD24 hrs), compared to the group with genistein treatment only (Figure 6a: E8 hrs + G24 hrs). Trypan blue staining showed consistent result that significant increase of cells losing homeostasis control in genistein treatment group in the presence of caspase inhibitor Z-VAD-fmk (Figure 6b). Together, these results implied that genistein could also influence other cellular recovery processes and disrupt the homeostasis maintenance of stressed cells to cause necrosis which is secondary to the primary apoptotic program.

## Discussion

Low concentration of ethanol has been reported to induce apoptosis in animal and cell line models [24-26]. Ethanol-inducible Cytochrome-P4502E1 (*CYPE* gene product) may contribute to the death signal by producing reactive oxygen species [27]. In this study, we optimized ethanol treatment condition in HeLa cell line. We provided evidence that ethanol-stressed HeLa cells displayed features including cell shrinkage, nucleus condensation, PS externalization, mitochondrial disruption and dysfunction, and caspase activation (Figure 1 and Figure 2e), which are characterized of apoptotic cell death [28-30]. Overall, the result that stressed HeLa cells showing apoptotic features could recover after stress removal is consistent with previous studies that loss of membrane asymmetry in early apoptosis [31,32] and the activation of caspase activity [33,34] by different apoptotic inducer were reversible. Reversible apoptotic event was also well characterized by time-lapse video microscopy in previous study, which showed that apoptotic dying cancer cells regained normal morphology and proliferate after the removal of inducer [34]. The reversibility of cancer cell from apoptosis raised interesting question whether apoptotic recovery contributes to survival or repopulation of cancer after cycles of chemotherapy.

Accumulating evidence suggested that cancer preventative agents might be combined with chemotherapy or radiotherapy to enhance treatment outcome [23]. Soybean isoflavone genistein is the one of the most widely studied compound for its prominent anti-proliferation effects on cancer cells [16,17]. In addition to its cytotoxicity and anti-proliferation effect, we showed that genistein could promote cell death of ethanol-stressed HeLa cells with apoptotic features (Figure 2). As genistein was reported to enhance the effects of anti-cancer drugs [16,17,21,22] and anti-cancer agents could induce apoptosis in cancer cells [3-5], the death-promoting effect of genistein on stressed HeLa cells may at least partially contribute to enhanced anti-tumor effect when it was combined with anti-cancer drugs.

Our results also demonstrated that the recovering HeLa cells were addicted to transcription and translation processes (Figure 3a), implying that cells undergoing recovery process were highly dependent on *de novo* synthesis. As the cleavage of cellular proteins by activated caspase is irreversible, it is anticipated that caspase activity should be counteracted and the damaged cellular proteins should be replenished if a cell is capable of recovering from caspase-dependent apoptotic event [35]. In this view, *de novo* synthesis may not only function to compensate for cellular damages, but also to clear the caspase activity. Our data showed the up-regulation of *XIAP* and *MDM2* genes after the stress removal in HeLa cell (Figure 3c). XIAP protein is a member of IAP (Inhibitors of Apoptosis Proteins) family, which not only directly interacts with activated caspases to block their activities but also functions as E3 ubiquitin ligase to assist the ubiquitination of activated caspases and their subsequent degradation [36,37]. MDM2 is a well-documented ubiquitin ligase binding to p53 to negatively regulate its stability and transcriptional activity [38,39]. The up-regulation of *XIAP* and *MDM2* may function to eliminate the caspase activity and terminate the p53-dependent apoptosis signal after stress removal. Indeed, inhibiting MDM2 or XIAP activity caused persistent caspase activity and enhanced cell death (Figure 4). In addition, *CREB1* gene that acts as a transcription factor for other pro-survival genes [40,41] was up-regulated during recovery phase (Figure 3c). Together, our data supports the hypothesis that both counter-apoptotic signals and pro-survivor signals are involved in the recovery process from ethanol-induced apoptotic events.

Genistein exerted obvious interference on the expression profiles of *ATF3*, *XIAP*, *MDM2*, *TP53*, *NOXA* and *PUMA* genes in HeLa cell (Figure 3c). *ATF3* gene encodes a rapidly-induced stress sensor that functions to stabilize p53 protein and contribute to the acceleration of pro-apoptotic signal [42,43]. Our data showed that genistein treatment delayed the down-regulation of *ATF3* after the removal of stress (Figure 3c). Together with attenuated *MDM2* expression level, delayed down-regulation of *ATF* by genistein may function to increase p53 level and the subsequent trans-activation of p53 target genes *NOXA* and *PUMA* (Figure 3c), which encode the pro-apoptotic proteins Noxa and Puma [44,45]. Interestingly, genistein also delayed the down-regulation of anti-apoptotic gene *MCL1* (Figure 3c; ref. [46]). However, this anti-apoptotic signal may be rapidly overwhelmed by the up-regulation of *NOXA* and *PUMA* genes.

Now that anti-apoptotic genes *XIAP* and *MDM2* were up-regulated during the recovery phase and genistein suppressed their up-regulation (Figure 3c), and XIAP or MDM2 inhibitor caused persistent caspase activity in the recovering cells (Figure 4d, e), the death-enhancing effects of genistein may result from the hindrance to the termination of apoptotic effectors. However, the addition of caspase inhibitor did not rescue the cells from genistein’s inhibitory effects (Figure 5). Genistein together with caspase inhibitor increased the percentage of necrotic events in the cells recovering from apoptosis (Figure 6). Previous studies have demonstrated that genistein could suppress the AKT and NF-κB pro-survivor signaling pathway in various type of cancer cells [21,47-49]. AKT- NF-κB pro-survivor signaling pathway was reported to up-regulate anti-apoptotic genes like *XIAP*[50,51]. AKT also functioned to activate and enhance MDM2 activity [52,53]. Indeed, previous researchers have showed that genistein down-regulated MDM2 at transcriptional and post-translational level [54] and reduced XIAP protein was detected following genistein treatment [55]. In this view genistein’s death-promoting action on the cellular recovery may result from both the persistent pro-apoptotic signal and the impaired recovering processes due to the impairment of pro-survival pathway in HeLa cell. However, it remains to be clarified how the recovering cells diverted the effect of genistein to necrotic death in the presence of caspase inhibitor.

**Figure 7:**
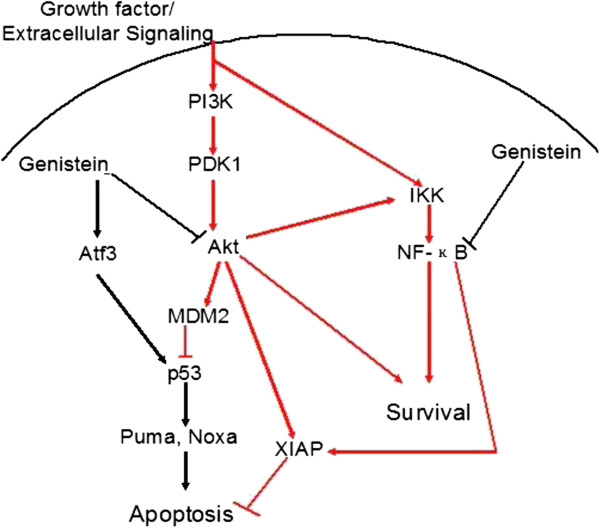
**Schematic illustration of the mechanism of genistein’s inhibition on the recovery from apoptotic event.** Black lines indicate pro-apoptotic signals; red lines indicate pro-survival signals.

Collectively, on the basis of our results and previous studies, we propose a hypothetic pathway through which genistein may exert its death-promoting effect on the stressed HeLa cells (Figure 7). Possibly through inhibiting the activity of AKT and NF-κB, genistein suppresses the up-regulation of *XIAP* and *MDM2* that were showed to be involved in the recovery process. Genistein treatment also sustains *ATF3* expression level after the removal of stress, which together with decreased *MDM2* level contributes to the stabilization of p53. p53 accumulation trans-activates its target genes including *NOXA* and *PUMA* to cause persistent pro-apoptotic signals. Since AKT pathway functions to promote protein synthesis, regulate glucose metabolism, suppress pro-apoptotic signal and activate NF-κB [56,57], the attenuated AKT and NF-κB activity due to genistein treatment may weaken the ability to antagonize existing apoptotic effectors and impair the recovery from the damages insulted by the stress.

## Conclusions

In summary, this study revealed novel effects of genistein on inhibiting the recovery of HeLa cells from apoptotic events. This effect of genistein on other types of cancer cells and related mechanisms remain to be elucidated, and it is possible that this effect could account for the enhanced anti-tumor effect when genistein is combined with chemotherapeutic drugs. It is anticipated that the discovery of more effective inhibitor of .apoptotic recovery could help to improve chemotherapy.

## Methods

### Cell lines and cell culture

Human cervical cancer cells HeLa (from ATCC) was cultured in DMEM (Dulbecco’s minimum essential medium, Gibco). The medium was supplemented with 10% fetal bovine serum (Hyclone, Thermo Scientific), 100 U/ml penicillin and 100 μg/ml streptomycin (Gibco, Carlsbad, CA, USA) at 37°C under humidified atmosphere of 5% CO2/95% air. Cells were seeded on tissue culture plates until cell density reached 60% to 70% confluence before being subjected to each experiment. The compounds used for testing the death-promoting effect on stressed cancer cell includes: Genistein (Cayman Chemical, Ann Arbor, MI, USA), XIAP inhibitor Embelin (Sigma-Aldrich, St. Louis, MO, USA), MDM2 inhibitor boranyl-chalcone (Calbiochem, La Jolla, CA, USA), General caspase inhibitor Z-VAD-fmk (MP Biochemical, Aurora, Ohio, USA), Actinomycin D and Cycloheximide (Sigma-Aldrich).

### Live cell staining and imaging

Cells were seeded on a glass coverslip (Marienfeld, Lauda-Ku¨nigahofen, Germany) in dish (Nunc, 60 mm). Mitochondria was stained with 50 nM MitoTracker Red CMXRos (Invitrogen, Eugene, Oregon, USA) for 45 minutes and nuclei were stained with 250 ng/ml Hoechst 33342 (Invitrogen) for 15 minutes. Then the cells were washed three times with PBS and then cultured in CO_2_-independent medium (Invitrogen) on a thermo-cell culture FCS2 chamber (Bioptechs, Butler, PA,USA) that was mounted onto adapter in the stage of an inverted fluorescence microscope Cell Observer (Carl Zeiss, Jena, Germany). Cell morphology was visualized by differential interference contrast (DIC) channel. Mitochondria and nucleuses were detected by fluorescence excited at 561 and 405 nm respectively. Cell images were captured with a monochromatic CoolSNAP FX camera (Roper Scientific, Pleasanton, CA, USA) with x63 numerical aperture (NA) 1.4 Plan-Apochromat objective (Carl Zeiss), and analyzed by AxioVision 4.2 software (Carl Zeiss).

### MTT cell viability assay

Cells were seed for 24 h on flat bottomed plates (Nunc, 96 well). After different treatments, 16 μL of 3-(4,5-dimethylthiazol-2-yl)-2,5 -diphenyltetrazolium bromide (MTT) reagent (5 mg/ml, Sigma-Aldrich) was added to each well and incubated at 37°C for 3 h. After incubation, culturing medium was aspirated and 120 μl DMSO (Sigma-Aldrich) was added to each well for 10 minutes’ incubation at room temperature. Absorbance was measured at a wavelength of 570 nm (reference wavelength 650 nm) by SpectraMax 250 microplate reader (Molecular Devices Corp, Concord, ON, Canada).

### BrdU cell proliferation assay

The cell proliferation ELISA, BrdU kit (Roche) was used according to the manufacturer’s instructions. Cells were seeded into 96-well plates (5 × 10^3^ cells / well) and treatments were added the next day. After each treatment, cells were labeled with a 10 mM BrdU solution (5′bromo-2′deoxyuridine) in culture medium and incubated for an additional 48 h at 37°C. The medium was then replaced with 100 ul Fixative/Denaturing solution and incubate for 30 minutes at room temperature. Anti-BrdU Antibody in 1:1000 dilutions was added to each well and the plates were incubated at room temperature for 1 h. After three washing steps, peroxidase-substrate color development solution (100 μl) was added to each well and the color reaction was stopped after 10 minutes with 25 μl 1 M H_2_SO_4._ Absorbance of samples was measured at 370 nm (reference wavelength 492 nm).

### LDH Cytotoxicity Assay

The cell death LDH cytotoxicity detection kit (Roche) was used according to the manufacturer’s instructions Briefly, cells (1 × 10^4^/well) were seeded in 96-well plates in 100 μl of medium and incubated at 37°C for 24 h before treatment. The maximum LDH release (high control) from cells was determined by the addition of 2% 100 ul Triton X-100 (Sigma-Aldrich), followed by incubation at 37°C for 1 h. The minimum LDH release was determined from untreated cells. After centrifugation at 200 g, supernatants of samples with different treatments were transferred to a new 96-well plate, and the reaction mixture from the kit was added. After incubate for 30 minutes at room temperature, absorbance at 490 nm (reference wavelength 620 nm) was recorded using microplate reader. ABS (absorbance) experiment = (mean absorbance from the treated cells) - (absorbance from blanks); ABS low = (mean absorbance from low control, untreated cells) - (absorbance from blanks); ABS high = (mean absorbance from high control, Triton X-100 treated cells) - (absorbance from blanks); the percentage of cytotoxicity was calculated as: (Abs experiment – Abs low)/(Abs high – Abs low).

### Quantitative Real-Time PCR

RNA was extracted using the RNeasy Mini Kit (Qiagen) according to the manufacturer’s protocol. RNA concentrations were measured spectrophotometrically. First-strand cDNA was synthesized from 0.1 μg RNA template using Reverse Transcriptase Polymerase Chain Reaction. RT-qPCR amplifications were conducted on BioRad iQ™5 Multicolor Real-time PCR Detection System (Biorad), using SYBR® Green qPCR Supermix (Invitrogen). Triplicate reactions were performed for each sample, both for target and reference gene. The expression level of each gene was determined by absolute quantification method. First, the absolute value of each gene was determined by standard curve. In each sample, the value of each target gene was divided by the value of *GAPDH* reference gene for normalization. The normalized data were expressed as normalized mRNA level (the ratio of target gene level to reference gene level). The Primers used for RT-qPCR are listed in Table 1.

**Table 1:** Primer sequences for target genes

**Gene symbol**	**Forward Primer (5’ to 3’)**	**Product size**
	**Reverse Primer (5’ to 3’c**	
***BCL2***	ATGTCCAGCCAGCTGCACCTGAC	319 bp
	GCAGAGTCTTCAGAGACAGCCAGG	
***MDM2***	CCCTGGTTAGACCAAAGCCAT	190 bp
	GGCACGCCAAACAAATCTCC	
***GAPDH***	CATGAGAAGTATGACAACAGCCT	113 bp
	AGTCCTTCCACGATACCAAAGT	
***XIAP***	GGGTTCAGTTTCAAGGACATTAAG	182 bp
	CGCCTTAGCTGCTCTTCAGTAC	
***ATF3***	AACCTGACGCCCTTTGTCAAG	138 bp
	TACCTCGGCTTTTGTGATGGA	
***BAX***	GATGCGTCCACCAAGAAGCT	170 bp
	CGGCCCCAGTTGAAGTTG	
***MCL1***	TAAGGACAAAACGGGACTGG	137 bp
	ACCAGCTCCTACTCCAGCAA	
***TP53***	GAGGTTGGCTCTGACTGTACC	133 bp
	TCCGTCCCAGTAGATTACCAC	
***CREB1***	TTAACCATGACCAATGCAGCA	140 bp
	TGGTATGTTTGTACGTCTCCAGA	
***PUMA***	GACCTCAACGCACAGTACGAG	98 bp
	AGGAGTCCCATGATGAGATTGT	
***NOXA***	ATGCCTGGGAAGAAGGCGC	164 bp
	CAGGTTCCTGAGCAGAAGAGT	

### Western blot

Proteins collected from cell lysate were separated on a 10-15% SDS-PAGE gel and then transferred onto the Immun-Blot PVDF membrane (Biorad) using semi-dry transfer system (Biorad). After blocking, membrane was incubated overnight at 4°C with 1:1000 primary antibodies (anti-caspase 3, anti-PARP antibody from Cell Signaling, Beverly, MA USA; anti-caspase 7, anti-caspase 9 and anti-β-tubulin antibody from Santa Cruz Biotechnology, CA, USA). After washing three times with TBST, the membrane was incubated in corresponding horseradish peroxidase- conjugated secondary antibody (Bio-Rad, Hercules, CA, USA) at 1:5000 dilution for another 1 h. The excessive antibody was washed away three times with TBST and signal was developed with ECL western blotting detection system (Amersham Biosciences).

### Annexin V/ Propidium Iodide Assay

After each treatment, cells were collected by trypsinization and washed with chilled PBS. The cells were stained with an Alexa Fluor® 488 Annexin V/Dead Cell Apoptosis Kit (Invitrogen, Eugene, Oregon, USA) according to manufacturer’s instruction. Briefly, cell density was determined and 1×10^6^ cells were suspended in 1 ml 1× annexin binding buffer, followed by addition with 10 ul Alexa Fluor® 488 Annexin V (component A) and 2 ul 100 μg/ml propidium iodide (component B) in the dark, at room temperature for 5 minutes, and immediately analyzed by flow cytometry (Cytomics FC500, Beckman Coulter, CA, USA). Cells Annexin V-positive/ PI-negative were considered as early apoptotic, cells doubly positive as late apoptotic or necrotic.

### Trypan Blue Dye Exclusion Assay

Cells were washed with PBS twice and detached using 0.25% /EDTA Trypsin (Gibco). Cells were collected by centrifuge at 200 g for 5 minutes and the supernatant was discarded. The pellet was re-suspended in 1 ml PBS, and 1 part of 0.4% trypan blue (Gibco) was mixed with 1 part cell suspension. 10ul trypan blue/cell mixture was added to hemacytometer (Hausser Scientific, Horsham, PA, USA) for quantification. The unstained (viable) and stained (nonviable) cells were counted separately.

### Cleaved-Caspase 3 Immunostaining

Cells (1×10^6^/mL) were collected and fixed in 4% para- formaldehyde (Sigma-Aldrich) for 10 minutes at 37°C, following by permeabilization with 0.1% (v/v in fixative) Triton X-100 (Sigma-Aldrich) for 30 minutes at room temperature. After three times’ rinse with incubation buffer (0.5% BSA in PBS) by centrifuge, cells were incubated with 1:1600 rabbit anti-cleaved caspase-3 IgG (Cell Signaling, Beverly, MA) overnight at 4°C. After rinsing three times, cells were incubated with 1:1000 Alexa Fluor® 488-conjugated goat anti-rabbit IgG (Invitrogen, Eugene, Oregon, USA) solution with 250 ng/ml Hoechst 33342 (Invitrogen) for 1 h at room temperature. Then cells were rinsed with incubation buffer and collected by centrifuge. Cell pellet was re-suspended in 0.1 ml Anti-fade Buffer (Invitrogen). 0.01 ml of suspended cells was spread onto slides for fluorescence microscopy analysis.

### Statistical analysis

Each experiment was repeated three times for consistency of the result. The results were either expressed relative to controls, or as percentages of the cell population (mean ± SE, n = 3). IC50 value was calculated using GraphPad Prism 5 software. Significance of the differences in multiple mean values comparison was determined by one-way ANOVA when there is only one variable. Two-way ANOVA was used when there were two variables and *P* < 0.05 was considered to be statistically significant.

## Abbreviations

AKT: also known as Protein Kinase B (PKB); ATF3: Activating Transcription Factor 3; CREB1: CAMP Responsive Element Binding protein 1; MDM2: Murine Double Minute; NF-κB: Nuclear Factor kappa-light-chain-enhancer of activated B cells; PARP: Poly (ADP-ribose) Polymerase; PS: Phosphatidylserine; XIAP: X-linked Inhibitor of Apoptosis Protein.

## Competing interests

The authors declare that they have no competing interests.

## Author’s contributions

Conceived and designed the experiments: XX, MCF. Performed the experiments: XX, SSW; Analyzed the data: XX, SSW; Contributed reagents/materials/analysis tools: XX, SSW; Wrote the paper: XX, SSW, CSW; All authors read and approved the final manuscript.
